# Mental Health Profiles Based on Self-Regulation and Technology Use in the Digital Era in a Spanish-Speaking Sample: Latent Profile Analysis

**DOI:** 10.2196/77167

**Published:** 2026-02-11

**Authors:** Angélica Garzón Umerenkova, Marisleidy Alba Cabañas, Erika Andrea Malpica-Chavarria

**Affiliations:** 1 School of Psychology, Fundación Universitaria Konrad Lorenz, Bogotá, Colombia; 2 Business School, Fundación Universitaria Konrad Lorenz, Cra. 9 Bis #No. 62 - 43, Bogotá, Colombia, +57 1 3472311

**Keywords:** cyberpsychology, self-regulation, problematic technology use, smartphone addiction, nomophobia, Colombia, latent profile analysis

## Abstract

**Background:**

The widespread use of digital technologies has raised growing concerns about their impact on mental health. While self-regulation has been proposed as a protective factor, little is known about how distinct psychological profiles based on self-regulatory and technology use patterns relate to psychological distress. Person-centered approaches, such as latent profile analysis, may offer deeper insights, particularly in underrepresented populations.

**Objective:**

This study aimed to identify latent psychological profiles based on self-regulation, nomophobia (fear of being without a phone), and problematic use of the internet and social media (defined by behavioral symptoms), to examine their associations with general psychological distress and the presence of emotional symptoms in a Colombian sample. Additionally, the predictive roles of age and gender in class membership were explored.

**Methods:**

Participants were recruited through a convenience sampling strategy aimed at ensuring heterogeneity of the sample in terms of age and gender. A total of 453 participants aged 12 to 57 years (mean 21.03, SD 8.41 years; 257/453, 56.7% female) completed validated measures of self-regulation (Abbreviated Self-Regulation Questionnaire), nomophobia (Nomophobia Questionnaire), internet and social media use (MULTICAGE-TIC, a multidomain screening questionnaire based on the CAGE framework), and psychological distress (General Health Questionnaire-12). Latent profile analysis was conducted using standardized scores of continuous variables. Model fit was assessed using the Bayesian information criterion, entropy, and bootstrapped likelihood ratio test. Differences in psychological distress scores across latent classes were examined through variance analysis (ANOVA) and regression models. A multinomial logistic regression tested the predictive value of age and gender for class membership.

**Results:**

The optimal solution revealed 4 distinct latent profiles (entropy=0.85). Class 1 showed high self-regulation and low problematic technology use, displaying the lowest psychological distress scores. Class 2 presented moderate levels across all indicators but the highest level of psychological distress. Classes 3 and 4 showed mixed patterns. Class 3 (higher information and communication technology [ICT] use and lower self-regulation) exhibited lower distress than class 2, whereas class 4 (younger individuals with low self-regulation and moderately high ICT use) showed higher distress than class 3. Psychological distress differed significantly across profiles (ANOVA, *P*<.001). Age and gender predicted class membership. Older males were more likely to belong to class 1, and younger females were more likely to be classified into classes 3 and 4.

**Conclusions:**

Latent profile analysis identified distinct configurations of digital behavior, self-regulation, and psychological distress. Self-regulation consistently differentiated profiles with lower distress scores, suggesting its relevance for understanding how individuals manage ICT use. These findings support the value of person-centered approaches to characterize heterogeneous patterns of technology-related behaviors. The study provides evidence from a Spanish-speaking sample, offering a novel perspective on psychological distress and problematic technology use in contexts that remain underrepresented in the literature.

## Introduction

### Background

The global use of information and communication technologies (ICTs) has grown exponentially over the past decade, with more than 67% of the world’s population connected as of 2023 [1]. In Latin America, countries, such as Brazil, Argentina, and Colombia, report daily usage exceeding 9 hours, driven by the widespread adoption of mobile devices and the growing popularity of social media platforms [1-3].

In parallel with this digital expansion, studies have reported a rise in problematic behaviors associated with technology use, with documented consequences for both physical and psychological health [4]. Although terms, such as problematic use, digital addiction, and technology dependence, are frequently adopted, there is still no consensus regarding their clinical classification. The literature distinguishes subtypes, including problematic internet use, social media use, smartphone use [5], gaming disorder, and nomophobia [6], which reflect the multidimensional nature of the phenomenon [7]. Some authors argue that problematic technology use shares features with behavioral addictions, social anxiety, and sleep disturbances [8] or compulsive habits [9,10]. However, others suggest that its consequences do not reach the severity of substance addictions and therefore recommend distinguishing among abuse, dependence, and problematic use [11].

Among these, nomophobia has emerged as a relevant subtype of problematic technology use, with direct psychological effects (anxiety, stress, and insomnia). Defined as the irrational fear of being without a mobile phone [6,12], nomophobia is associated with anxiety, insomnia, dependency, and greater psychological vulnerability [13].

This raises the question of which personal protective factors can mitigate the consequences of problematic technology use. Self-regulation, understood as the ability to manage impulses and behaviors, has been studied as a protective factor against both substance-related and behavioral addictions. Evidence suggests that higher self-regulation is associated with greater psychological well-being, healthier digital habits, and reduced procrastination [14-16]. Baumeister and Heatherton [17] proposed that self-regulation failure occurs when individuals are unable to inhibit immediate impulses in favor of long-term goals. In the digital context, this translates to difficulty controlling screen time or compulsive smartphone use, despite the obvious negative consequences.

Empirical evidence supports the notion that deficits in self-regulation are linked to both problematic technology use and broader mental health problems [18-20]. Conversely, higher self-regulation is associated with greater psychological well-being, healthier digital habits, and reduced procrastination [21-24].

Most previous studies have addressed problematic technology use, focusing on average trends and assuming population homogeneity, which obscures subgroup differences. This gap may be explained by the predominant use of variable-centered approaches [25]. In contrast, person-centered approaches, such as latent profile analysis (LPA), enable the identification of specific profiles (subgroups characterized by combinations of self-regulation, ICT use, and nomophobia), thereby revealing differential patterns of psychological distress. For example, it has been demonstrated that self-regulation profiles among students were significantly associated with variations in well-being [26] and personality profiles influenced mental health differently during the pandemic [27].

### Aim

This study aimed to identify distinct psychological profiles through LPA, based on the levels of self-regulation, nomophobia, and internet and social media use. By adopting a person-centered approach, the study seeks to uncover subgroups characterized by combined patterns of problematic technology use and self-regulation.

The identified profiles will be examined in relation to age and sex as covariates to determine whether these sociodemographic variables predict membership in specific profiles. In addition, the profiles will be compared in terms of their differential levels of psychological distress, thereby providing a more detailed understanding of whether distinct patterns of problematic technology use and self-regulation have varying impacts on distress scores. This, in turn, provides a basis for designing differentiated and personalized interventions.

Finally, by incorporating a Colombian sample, this study contributes to addressing the scarcity of empirical evidence in Spanish-speaking populations, particularly in Latin America, where digital growth has not been matched by equal progress in preventive and clinical research [28].

## Methods

### Ethical Considerations

The project was previously approved by the Bioethics Committee of the Konrad Lorenz University Foundation (number: 07-23). This study was conducted in accordance with the ethical principles established in Law 1090 of 2006, particularly Article 13 [29], which states that all professional conduct in psychology must be based on the core principles of the profession, including beneficence and nonmaleficence. In line with these principles, the study used self-report instruments without applying procedures that could pose harm or risk to participants.

This consent process ensured participant autonomy by providing full study information, the opportunity to ask questions, and the option to participate voluntarily. Throughout the research process, participant responses were anonymized, and data were reported in aggregate form to prevent identification of individuals. All identifying data, such as gender, education level, and individual test responses, were replaced with codes. Participation in the study was voluntary and involved no financial compensation, and participants could withdraw at any time without consequences.

### Participants

Participants were recruited through a convenience sampling strategy aimed at ensuring heterogeneity of the sample in terms of age and gender, utilizing both digital and institutional channels. Specifically, the research team distributed the study invitation via social media platforms (Facebook and WhatsApp) and during in-person and virtual classes at 3 universities in Colombia. Individuals interested in participating accessed the study link hosted on Jotform, which included the eligibility criteria and the informed consent form. Recruitment was conducted directly by the research team members. The inclusion and exclusion criteria are presented in Textbox 1.

Textbox 1. **Inclusion and exclusion criteria.**

**Inclusion criteria**

- Age between 12 and 60 years
- Colombian nationality
- Possession of a smartphone with internet access
- Ability to provide informed consent

**Exclusion criteria**

- Diagnosed with a severe condition that limits the ability to answer self-report questionnaires
- Inability to read or understand Spanish
- Lack of internet connectivity that prevents completing an online survey
- Withdrawal of informed consent during participation

### Instruments

The MULTICAGE-TIC (a multidomain screening questionnaire based on the CAGE framework) [30] is a questionnaire designed to identify problematic behaviors associated with the use of ICT. Its design is based on the MULTICAGE CAD-4 [31], a screening instrument that assesses compulsive behaviors related to both substance and nonsubstance use and has been widely used to measure behavioral addictions. The MULTICAGE-TIC evaluates 5 areas of ICT use: mobile phones, internet, social media, video games, and instant messaging. A subscale with 4 items represents each area, with each scoring 4‐8 points. Responses are dichotomous (no=1 point; yes=2 points). According to the suggested interpretation of the authors, usage is classified as nonproblematic (when no response or 1 response is affirmative; 4‐5 points), at risk (when 2 responses are affirmative; 6 points), and problematic (when 3-4 responses are affirmative; 7‐8 points). For this study, the areas of internet use and social media use (each with 4 items) were considered representative of overall ICT usage. In previous studies, the instrument has demonstrated adequate reliability levels, with McDonald ω and Cronbach α coefficients above .70 across all subscales. Additionally, its scores have been found to correlate significantly with symptoms of prefrontal dysfunction and psychological distress [32].

The Nomophobia Questionnaire (NMP-Q) [6] measures nomophobia, which is defined as a situational phobia caused by a lack of availability of a smartphone or the thought of not having it, not being able to use it, or losing it. It contains 20 items and 4 dimensions: “communication inability” (6 items), “loss of connection” (5 items), “inability to access information” (4 items), and “renunciation of convenience” (5 items), which can explain 69.5% of the variance. The internal consistency of the factors is between 0.81 and 0.94. Responses are recorded on a 7-point Likert scale, ranging from “1” (strongly disagree) to “7” (strongly agree). The total score ranges from 20 to 140. The Spanish version of the test, which has been validated in a Colombian population [33], was used.

The General Health Questionnaire-12 (GHQ-12) is a brief screening scale designed to detect potential indicators of psychological distress and the risk of mental disorders in the general population [34]. The version used was validated for the Colombian population [35]. It assesses aspects such as emotional symptoms, stress, anxiety, and overall psychological functioning. The scale consists of 12 items with a 4-point Likert response format (range 0‐3). Higher scores on the GHQ-12 reflect greater psychological distress and poorer mental health. Previous studies have shown a unidimensional structure and significant associations with variables such as experiential avoidance and life satisfaction [35].

The Abbreviated Self-Regulation Questionnaire (CAR-abr) was initially developed by Brown [14]. Its abbreviated Spanish version, which has been validated in a Colombian population [15], was used. It assesses individuals’ general self-regulatory capacity. It comprises 17 items, with Likert-scale response options organized into 4 subscales. The scale has demonstrated solid psychometric performance in terms of both the fit to the Rasch model and the quality of its items and response categories. Previous studies have reported internal consistency values ranging from 0.75 to 0.80, as well as evidence of structural and content validity [15].

### Procedure

The project was previously approved by the Bioethics Committee of the Konrad Lorenz University Foundation (number: 07‐23). The protocol and consent are consistent with the Colombian Code of Ethics [29], which guarantees the anonymous and confidential treatment of the data collected. With institutional authorization, participants were recruited through social networks and public schools in the city. Participants provided informed consent before answering the questionnaires, and guardian assent was obtained for minors. The instruments were applied virtually and voluntarily, without remuneration.

### Data Analysis

All analyses were conducted in RStudio (version 2024.12.1+563; Posit).

To address the possibility of common method bias arising from same-source, same-time self-reports, we planned to conduct 2 complementary tests. First, we applied the Harman single-factor test using a polychoric exploratory factor analysis with all items (self-regulation, nomophobia, internet use, social media use, and GHQ-12 score). Second, we conducted a series of confirmatory factor analyses comparing the following three models: (1) a trait model specifying the 5 latent constructs, (2) a 1-factor model in which all items loaded on a single factor, and (3) an unmeasured latent method construct model that incorporated both the trait factors and a latent method factor. Analyses were performed using the packages psych (for polychoric exploratory factor analysis), lavaan (for confirmatory factor analysis estimation), and semTools (for reliability and method-factor modeling).

All psychological and behavioral indicators were included in an LPA, a person-centered modeling approach designed to identify subgroups of individuals based on similar patterns of responses [36]. The LPA was conducted using 4 continuous indicators: self-regulation, nomophobia, internet use, and social media use. These variables were standardized before analysis to ensure comparability. Models with 1-4 profiles were estimated using the tidyLPA package in R.

Multiple statistical criteria, including the Bayesian information criterion (BIC), Lo-Mendell-Rubin likelihood ratio test (bootstrapped likelihood ratio test [BLRT]), class size, posterior probability, and entropy, guided model selection. The 4-profile model showed the lowest BIC and acceptable entropy (0.85), supporting its selection as the optimal solution. This model yielded 4 distinct psychological profiles based on the interplay between digital behavior and self-regulation.

To evaluate between-group differences in psychological distress across profiles, we first conducted an ANOVA. Pairwise comparisons were examined using Tukey post hoc tests. Then, psychological distress scores were modeled using an ordinary least squares linear regression, with latent classes entered as dummy-coded predictors (class 1 as the reference category). Additionally, a multinomial logistic regression model was estimated, with age and gender as covariates, to predict latent class membership. All visualizations and analyses were performed in R using packages such as ggplot2, nnet, and tidyLPA. Given the exploratory nature of this study and the person-centered approach adopted, no a priori hypotheses were specified regarding the number or structure of latent profiles.

## Results

### Participant Characteristics

The study recruited 453 participants located in Colombia. Participants were 196 males (43.3%) and 257 females (56.7%), reporting an age between 12 and 57 years, with an average age of 21.03 years (SD 8.41 years). In terms of age distribution, 221 participants were between 12 and 17 years old, 145 were between 18 and 25 years old, and 87 were above 26 years old. Moreover, 70.2% (318/453) of participants were elementary and high school students, while the remaining 29.8% (135/453) had completed technical or professional studies. The majority were single (423/453, 93.4%) and resided in urban areas (417/453, 92.1%). Sociodemographic data are presented in Table 1.

**Table 1.** Sociodemographic data.

Variable		Value (N=453), n (%)	
Sex			
Female		257 (56.7)	
Male		196 (43.3)	
Marital status			
Single		423 (93.4)	
Married		30 (6.6)	
Income^a^			
1		18 (4.0)	
2		87 (19.2)	
3		291 (64.2)	
4		46 (10.2)	
5		11 (2.4)	
Education level^b^			
Elementary school		84 (18.5)	
High school		234 (51.7)	
Technical education		54 (11.9)	
Undergraduate education		58 (12.8)	
Postgraduate education		23 (5.1)	

a Lower income (1) to higher income (5).

b Education level indicates the final level of education.

### Psychometric Properties

We obtained internal consistency values. For internet use, the McDonald ω coefficient was 0.61, composite reliability (CR) was 0.76, and average variance extracted (AVE) was 0.45. The social media use scale yielded robust psychometric indicators, with a McDonald ω coefficient of 0.75, a CR of 0.86, and an AVE of 0.62, exceeding the recommended threshold.

The NMP-Q showed the strongest indicators among the instruments evaluated. The McDonald ω coefficient and CR were 0.96 and 0.97, respectively, and both were in the excellent range. Additionally, the AVE reached 0.62, indicating adequate convergent validity. Factor loadings were consistently high (0.65-0.91), confirming the internal coherence and explanatory power of the scale.

The total score of the CAR-abr was used as a global indicator of self-regulation, and the values obtained demonstrated high reliability (ω=0.89; CR=0.90), supporting its internal consistency. Nevertheless, the AVE was 0.35, which is below the minimum criterion, suggesting that items do not share enough common variance to ensure strong convergent validity. Despite this, the reliability level suggested that the measure is stable, although it may be multidimensional in its factorial structure.

The GHQ-12 demonstrated adequate internal consistency, with a total McDonald ω coefficient of 0.75 and a CR of 0.76. The AVE was 0.45, which is slightly below the recommended threshold of 0.50, indicating limitations in convergent validity. This pattern indicates that, although the scale is reliable, some items contribute less common variance, which may reflect the multidimensional nature of psychological distress symptoms.

### Common Method Bias

The Harman single-factor test showed that the first factor accounted for 25.78% of the variance, which is well below the conservative threshold of 50%‐60%, indicating the absence of a dominant single factor [37]. In the confirmatory factor analyses, the trait model demonstrated good fit (comparative fit index [CFI]=0.959; Tucker-Lewis index [TLI]=0.957), whereas the 1-factor model showed substantially poorer fit (CFI=0.853; TLI=0.847; root mean square error of approximation [RMSEA]=0.181; standardized root mean square residual=0.155). The unmeasured latent method construct model did not yield any meaningful improvement over the trait model (ΔCFI=0.0001; ΔRMSEA=0.0000), and the variance attributable to the method factor was only 3.1% [38]. Taken together, these results suggest that although a small amount of variance may be attributable to method effects, common method bias did not substantively distort the relationships among the study constructs.

### Descriptive Statistics of Indicator Variables

The variables used to estimate the latent profiles included self-regulation, nomophobia, internet use, and social media use. Self-regulation showed a mean score of 58.21 (SD 9.58), while the nomophobia score ranged from 20 to 140 points, with a mean of 61.58 (SD 27.95). For “internet use” and “social media use,” usage has been classified as nonproblematic (when no response or 1 response is affirmative; 4‐5 points), at risk (when 2 responses are affirmative; 6 points), and problematic (when 3-4 responses are affirmative; 7‐8 points). Details are provided in Table 2.

**Table 2.** Descriptive statistics.

Variable	Mean (SD)		Minimum value	Maximum value
Self-regulation	58.21 (9.58)		25	84
Nomophobia	61.58 (27.95)		20	140
Internet use	5.86 (1.30)		4	8
Social media use	5.45 (1.41)		4	8

### Associations Between Variables

Following previous guidelines [39], correlations below 0.30 were considered small, those at 0.30-0.50 were considered moderate, and those above 0.50 were considered large. In psychological and health sciences, even small associations may be meaningful at the population level [40]. The results of the associations were in the expected direction. Correlations between variables revealed moderate relationships between nomophobia and internet use (*r*=0.378) and social media use (*r*=0.361). There were significant negative correlations between self-regulation and internet use (*r*=−0.314), and between self-regulation and social media use (*r*=−0.211). Moreover, higher levels of self-regulation were associated with lower use of these ICTs.

Higher GHQ-12 scores indicated more psychological distress. A negative moderate correlation was found between self-regulation and psychological distress scores (*r*=−0.506), suggesting that higher self-regulation was associated with lower psychological distress. Nomophobia showed a small positive correlation with psychological distress (*r*=0.261). Moreover, there were positive correlations between internet use and psychological distress (*r*=0.327), and between social media use and psychological distress (*r*=0.188) (Table 3), indicating that more problematic technology use correlates positively with higher distress scores.

**Table 3.** Correlation values between variables.

Pair	Correlation	Bootstrapped 95% CI^a^	
Self-regulation versus nomophobia	−0.208^b^	−0.301 to −0.113	
Self-regulation versus internet use	−0.314^b^	−0.405 to −0.218	
Self-regulation versus social media use	−0.211^b^	−0.310 to −0.114	
Self-regulation versus psychological distress	−0.506^b^	−0.564 to −0.442	
Nomophobia versus internet use	0.378^b^	0.290 to 0.465	
Nomophobia versus social media use	0.361^b^	0.270 to 0.445	
Nomophobia versus psychological distress	0.261^b^	0.164 to 0.361	
Internet use versus social media use	0.592^b^	0.519 to 0.658	
Internet use versus psychological distress	0.327^b^	0.238 to 0.411	
Social media use versus psychological distress	0.188^b^	0.086 to 0.285	

a With 1000 iterations in the percentile method.

b Correlation is significant (*P*<.001; 2-tailed).

### LPA (Model Without Covariates)

An LPA was conducted to test solutions ranging from 1 to 4 classes. The 4-class model was identified as the best fit based on the BIC. Models with 1 to 4 classes were evaluated using fit indices such as the BIC, entropy, and the BLRT.

The 4-class model showed the lowest BIC (4710), an acceptable entropy (0.85), and a significant improvement over the 3-class model (BLRT *P*<.01), indicating adequate class separation. Additionally, all classes represented more than 5% of the sample (minimum=17%), suggesting a stable and well-differentiated solution. Details are provided in Table 4.

**Table 4.** Fit statistics and class distribution for competing latent profile analysis models.

Class model^a^	LogLik^b^	AIC^c^	BIC^d^	SABIC^e^	Entropy	BLRT^f^ *P* value	n_min (%)^g^	n_max (%)^h^
1	−2569	5154	5187	5161	1.000	—^i^	100	100
2	−2391	4807	4861	4819	0.817	.0099	34.4	65.6
3	−2356	4748	4822	4765	0.745	.0099	26.5	43.0
4^j^	−2285	4615	4710	4637	0.852	.0099	17.2	36.9

a Number of latent profiles or classes estimated in the model.

b LogLik: log-likelihood.

c AIC: Akaike information criterion.

d BIC: Bayesian information criterion.

e SABIC: sample size–adjusted BIC.

f BLRT: bootstrapped likelihood ratio test.

g Percentage of the sample assigned to the smallest class.

h Percentage of the sample assigned to the largest class.

i Not applicable.

j Selected model.

The entropy value of the 4-class model was 0.852, indicating good class separation. The BLRT yielded *P*<.01 for model comparisons, supporting the inclusion of additional classes.

### Description of the Resulting Profiles or Classes

**Figure 1.**
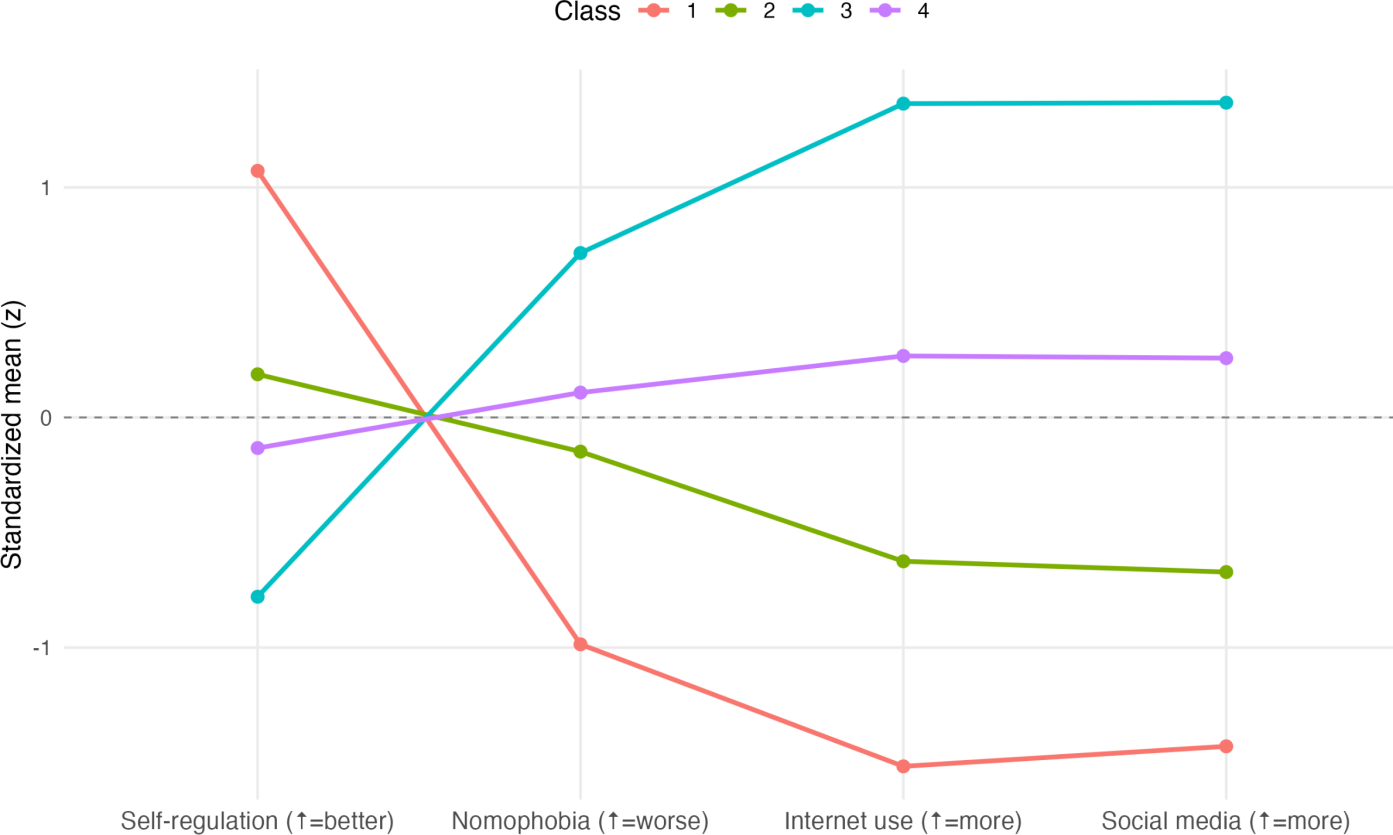
Four-class solution of the latent profile analysis (*z* scores across study variables).

Figure 1 presents the standardized mean scores (*z* scores) for each latent profile, representing the deviation of each class mean from the overall sample mean. For instance, class 1 scored about 1 SD above the sample mean in self-regulation (+1.0 SD) while remaining nearly 1 SD below the mean in nomophobia and ICT use (–1 SD). In contrast, class 2 showed slightly below-average scores across all variables, including self-regulation, nomophobia, and ICT use (–0.3 to –0.5 SD). Class 3 profiles had self-regulation about 1 SD below the mean (–1 SD), and nomophobia and ICT use more than 1 SD above the mean (+1 to +1.5 SD). Finally, class 4 exhibited moderately low self-regulation (–0.5 SD) in combination with average levels of ICT use and nomophobia (0 SD).

The qualitative interpretation of the latent classes suggested that class 1 was characterized by high self-regulation, low nomophobia, and low ICT use. Class 2 exhibited a more balanced but generally lower profile, characterized by slightly below-average levels of self-regulation, nomophobia, and ICT use. Class 3 was characterized by low self-regulation combined with high nomophobia and ICT use. Finally, class 4 presented a mixed profile, with moderately low self-regulation and average levels of nomophobia and ICT use. These standardized differences highlight that the most distinctive contrast was present between class 1 (high regulation, low ICT use) and class 3 (low regulation, high ICT use), whereas classes 2 and 4 represented more intermediate or mixed profiles.

### Analysis With Predictor Covariates (Age and Sex)

A multinomial model with covariates was also used. Age (as a continuous variable) and sex (1=female; 2=male) were included as latent class membership predictor covariates (class 1 as reference; see Table 5). Age significantly increased the odds of belonging to class 4 (β=−0.70, SE=0.20; *z*=−3.43; *P*<.001; odds ratio [OR] 0.50, 95% CI 0.34-0.74), indicating that younger participants were more likely to belong to this profile. The association with class 3 had a *P* value of .07, and no effect was observed for class 2 (*P*=.55). Regarding gender, males were less likely than females to belong to class 2 (β=−0.68, SE=0.26; *z*=−2.67; *P*=.007; OR 0.51, 95% CI 0.31-0.83). No other gender contrasts reached statistical significance (*P*>.29). Overall, demographic covariates showed selective but meaningful differentiation of latent profiles.

**Table 5.** Results of the multinomial model with age and sex as predictors of latent class membership.

Comparison (reference: class 1)	Predictor^a^	β (log-odds, standardized)^b^	SE	*z*	*P* value	OR^c^ (95% CI)	
Class 2 versus class 1	Age_z	−0.068	0.115	−0.59	.55	0.93 (0.75-1.17)	
Class 2 versus class 1	Male versus female	−0.680	0.255	−2.67	.007	0.51 (0.31-0.83)	
Class 3 versus class 1	Age_z	−0.252	0.137	−1.84	.07	0.78 (0.59-1.02)	
Class 3 versus class 1	Male versus female	−0.277	0.263	−1.06	.29	0.76 (0.45-1.27)	
Class 4 versus class 1	Age_z	−0.695	0.203	−3.43	<.001	0.50 (0.34-0.74)	
Class 4 versus class 1	Male versus female	0.277	0.280	0.99	.32	1.32 (0.76-2.28)	

a Age was z-standardized (mean 0, SD 1); sex was coded as 1=female and 2=male.

b Coefficients represent multinomial log-odds relative to class 1.

c OR: odds ratio.

### External Validation Using the Psychological Distress Variable

An ANOVA test was conducted to evaluate whether there were significant differences in psychological distress scores across the latent classes identified in the LPA model (Table 6).

**Table 6.** ANOVA results.

Source	Degrees of freedom	Sum of squares	Mean square	*F* test (*df*)	*P* value
Class (factor)	3	2212	737.4	18.32	.001^a^
Residuals	449	18,069	40.2	—^b^	—

a Statistically significant differences in psychological distress levels across the latent classes.

b Not applicable.

Subsequently, a Tukey post hoc test was performed to examine pairwise differences (Table 7).

**Table 7.** Tukey post hoc test results.

Class comparison	Mean difference (est.)	95% CI		Adjusted *P* value^a^
Class 2 versus class 1	5.08	3.09 to 7.06		<.001^b^
Class 3 versus class 1	1.68	−0.43 to 3.79		.17
Class 4 versus class 1	4.62	2.38 to 6.86		<.001^b^
Class 3 versus class 2	−3.40	−5.68 to −1.12		<.001^b^
Class 4 versus class 2	−0.46	−2.86 to 1.95		.96
Class 4 versus class 3	2.94	0.44 to 5.45		.01^c^

a *P* values were adjusted using the Tukey method.

b *P*<.001.

c *P*<.05.

The Tukey test results indicated that class 2 differed from class 1 by an average of 5.08 points in psychological distress scores, suggesting that class 2 experienced more distress. Class 4 showed an average difference of 4.62 points compared with class 1, indicating high psychological distress. Class 3 had lower distress than class 2, with a significant difference of –3.40 points. Additionally, class 4 exhibited more distress than class 3, with a significant difference of 2.94 points. Pairwise comparisons confirmed differences across classes.

Finally, a regression model was also estimated to predict psychological distress based on latent class membership (Table 8).

**Table 8.** Regression model results^a^.

Coefficient^b^	Estimate	SE	*t* test (*df*)	*P* value
Intercept (class 1)	10.35	0.49	21.09 (449)	<.001^c^
Class 2	5.08	0.77	6.59 (449)	<.001^c^
Class 3	1.68	0.82	2.05 (449)	.04^d^
Class 4	4.62	0.87	5.31 (449)	<.001^c^

a Higher values reflect greater psychological distress (General Health Questionnaire-12).

b Adjusted *R*²=10.3%. Coefficients indicate mean differences in distress scores relative to class 1.

c *P*<.001.

d *P*<.05.

**Figure 2.**
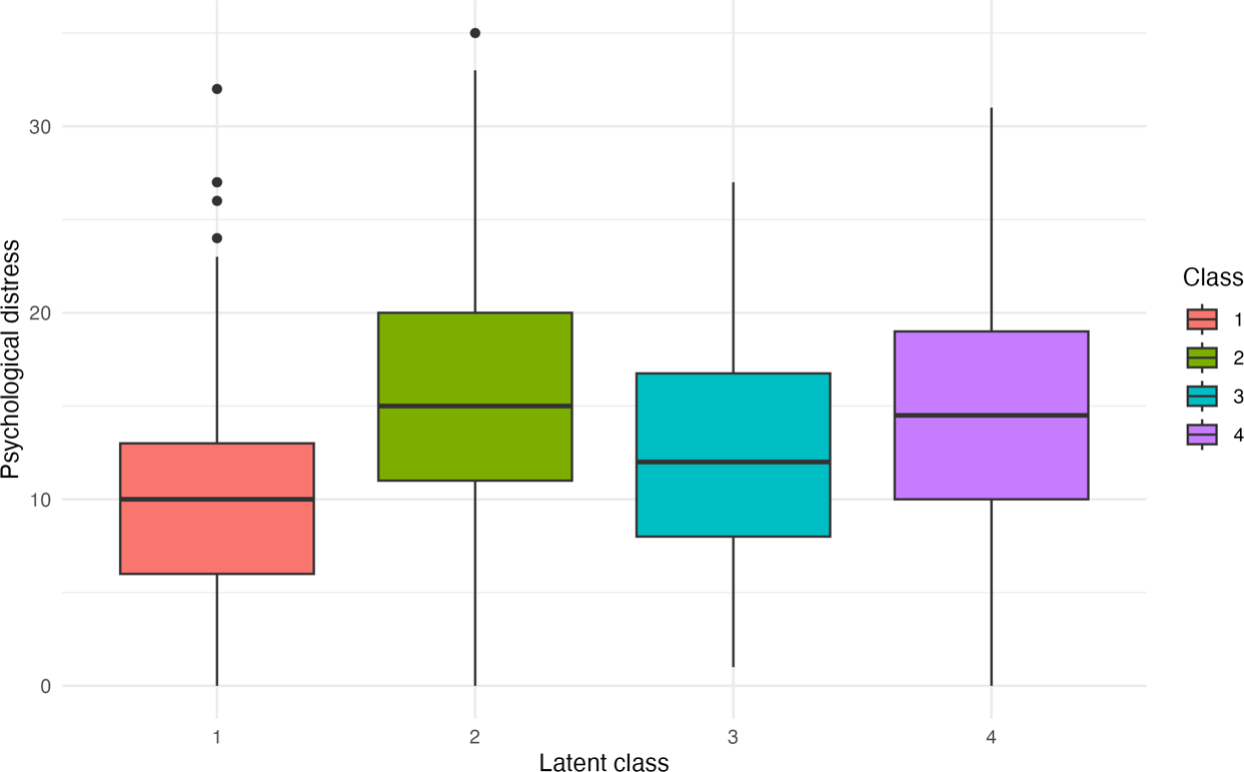
Psychological distress comparison across latent profile analysis classes.

Classes 2 and 4 showed higher psychological distress scores than class 1, indicating more average psychological symptoms (since higher scores on this scale reflect increased symptoms or distress). Class 3 showed a slight deterioration, although the effect was more moderate. Details are presented in Figure 2.

The results of combining the 4 classes, along with age, sex, and psychological distress, are shown in Table 9.

**Table 9.** Integrated results of profiles (classes 1‐4), age, sex, and psychological distress.

Class	Nomophobia	Problematic technology use	Self-regulation	Age^a^	Sex^a^	Psychological distress
1	Low (–1 SD)	Low (–1 SD)	High (+1 SD)	Balanced distribution	More men ↑	Lowest psychological distress
2	Below average (–0.3 to –0.5 SD)	Below average (–0.3 to –0.5 SD)	Below average (–0.3 to –0.5 SD)	Balanced distribution	More women ↑	Highest psychological distress
3	High (+1 to +1.5 SD)	High (+1 to +1.5 SD)	Low (–1 SD)	Younger­	More women ↑	Significantly lower distress than class 2; not significantly different from class 1
4	Average (0 SD)	Average (0 SD)	Moderately low (–0.5 SD)	Younger ↑↑	More women ↑↑	Significantly higher distress than classes 1 and 3

a Arrows indicate relative predominance in group composition based on descriptive comparisons. A single arrow indicates a higher proportion relative to other classes, whereas a double arrow indicates a more pronounced predominance. These symbols are descriptive and do not represent statistical significance or effect size.

## Discussion

### Principal Results

This study contributes to the field of cyberpsychology by providing empirical evidence on psychological profiles associated with problematic technology use and their relationship with psychological distress. Through a person-centered approach, we identified differentiated profiles characterized by distinct patterns of self-regulation, nomophobia, and ICT use, highlighting both consistent associations and apparent contradictions with prior research.

The LPA identified 4 distinct classes that significantly differed in distress scores. Class 1, which was characterized by high self-regulation and low problematic technology use, showed the lowest psychological distress. In contrast, classes 2 and 4 exhibited higher psychological distress, which was significantly worse than that of class 1. Class 2 was especially relevant because, despite reporting relatively low ICT use and moderate-to-low nomophobia, it exhibited the highest psychological distress, suggesting that other unmeasured factors may contribute to this vulnerability. Meanwhile, class 4, involving the youngest individuals, showed low self-regulation combined with high ICT use and nomophobia, also resulting in high psychological distress. Interestingly, the distress in class 3 was significantly lower than that in class 2, was comparable to that in class 1, and was lower than that in class 4. These findings indicate that the relationship between problematic technology use and distress is not monotonic, and the interplay of multiple factors must be considered.

### Comparison With Prior Work

There are some important aspects to consider. First, the results confirm that self-regulation is a central protective factor for mental health, even in the presence of high ICT use or nomophobia. This is consistent with studies indicating that it is not the absolute amount of use that explains psychological distress, but rather individuals’ ability to regulate their digital behaviors [41,42]. Accordingly, class 1, with high self-regulation, showed the lowest psychological distress, while profiles with low self-regulation (classes 3 and 4) experienced greater distress.

Second, class 2 concentrated on individuals with the highest psychological distress despite lower ICT use and lower levels of nomophobia. This apparently counterintuitive finding suggests that other factors not assessed in this study, such as neuroticism, loneliness, low self-esteem, lack of social support, and dysfunctional coping strategies [43-45], may influence the outcomes. The identification of this subgroup underscores the value of LPA, which, unlike variable-centered approaches, can reveal hidden profiles and inform the design of tailored interventions.

Third, the results suggest that age plays a protective role, but it is not linear. While prior research has emphasized adolescents and young adults as especially vulnerable to problematic social media use and nomophobia [46,47], our findings reveal nuances. Class 3, which consisted mainly of young adults with low self-regulation and high ICT use, did not show severe psychological distress, while class 4, which also consisted of younger individuals with a similar usage pattern, showed psychological distress. One possible explanation is that, in class 3, intensive ICT use may have been socially normalized in the age groups, reducing its negative effects [48,49]. In contrast, class 4 combined low self-regulation with more compulsive or maladaptive usage patterns, resulting in significantly more distress.

Fourth, gender showed selective effects. Men were less likely to belong to class 2 compared with class 1, while women were proportionally more represented in classes 3 and 4. These profiles combined higher ICT use and higher nomophobia, which aligns with previous research reporting higher levels of nomophobia and smartphone-related stress among women [49,50].

Finally, the profile comparison reinforces that problematic technology use is a multifactorial phenomenon that cannot be explained solely by usage intensity. A central contribution of this study is the analysis of a Colombian sample, which remains underrepresented in the international literature, thereby expanding evidence from Latin American contexts. A notable finding is that class 2 exhibited the highest psychological distress despite moderate ICT use, suggesting that additional individual or contextual factors—beyond usage frequency—may contribute to psychological outcomes. In addition, young women showed higher levels of nomophobia, greater intensive social media use, and a statistical association with increased psychological distress.

In this context, the role of nomophobia deserves attention. Although initially conceptualized as an emerging phenomenon linked to negative psychological outcomes, the profiles identified in this study suggest that its effects may not operate in a linear manner. For instance, class 3 showed high nomophobia and intensive ICT use combined with only moderate distress levels. This pattern suggests that nomophobia may interact with individual characteristics or contextual factors rather than exerting uniform effects across users. Understanding nomophobia as a component within broader behavioral configurations may help explain why similar levels of ICT engagement are associated with different psychological outcomes across groups.

### Limitations

Among the main limitations of this study is the exclusive use of self-report measures, which may have caused bias in the assessment of both technology use and psychological distress. To evaluate common method bias, we conducted the Harman single-factor test and a series of confirmatory factor analyses, which suggested minimal common method bias effects. Nevertheless, future research should incorporate external corroborating measures or social desirability scales to enhance validity.

Although the sample was diverse, a more representative sample of older adults would improve the generalizability of the findings. Additionally, longitudinal designs are necessary to establish causal relationships among the studied variables. Future studies should also incorporate qualitative methodologies to complement quantitative data and evaluate interventions that strengthen self-regulation as a preventive strategy in digital mental health.

Moreover, other psychological and contextual factors, such as resilience, coping strategies, social support, and specific motivations for technology use, should be considered in future analyses, as they may provide valuable insights into psychological vulnerability to problematic technology use [51]. Cross-cultural comparative studies are also recommended, as the dynamics of ICT use and its mental health implications may vary depending on sociocultural contexts. Complementing self-reports with objective indicators (eg, screen time and activity logs) can be considered to enhance measurement accuracy. Ultimately, analyzing the differentiated impact of various ICT types, such as social media, video games, and educational platforms, may provide further insights into their distinct implications for psychological well-being.

### Conclusions

The results of this study offer a comprehensive, data-driven perspective on how different patterns of technology use and personal resources shape distinct psychological risk profiles. Strengthening self-regulation has emerged as a promising pathway to promote healthier ICT use, particularly among younger populations and within educational or community contexts.

Our findings underscore the need for differentiated and personalized interventions that enhance self-regulation and promote adaptive technology use, with special attention to vulnerable groups, such as class 2 (individuals with high psychological distress despite low ICT use) and class 4 (young individuals with low self-regulation and high nomophobia). Future studies should expand this framework by incorporating additional psychological and contextual factors, such as social support, neuroticism, and coping strategies, to better explain heterogeneity in the psychological consequences of technology use patterns.

## Abbreviations

AVE: average variance extracted
BIC: Bayesian information criterion
BLRT: bootstrapped likelihood ratio test
CAR-abr: Abbreviated Self-Regulation Questionnaire
CFI: comparative fit index
CR: composite reliability
GHQ-12: General Health Questionnaire-12
ICT: information and communication technology
LPA: latent profile analysis
NMP-Q: Nomophobia Questionnaire
OR: odds ratio
RMSEA: root mean square error of approximation
TLI: Tucker-Lewis index
